# Family Risk Factors Associated With Oppositional Defiant Disorder Symptoms, Depressive Symptoms, and Aggressive Behaviors Among Chinese Children With Oppositional Defiant Disorder

**DOI:** 10.3389/fpsyg.2019.02062

**Published:** 2019-09-24

**Authors:** Xiuyun Lin, Yanbin Li, Shousen Xu, Wan Ding, Qing Zhou, Hongfei Du, Peilian Chi

**Affiliations:** ^1^Institute of Developmental Psychology, Beijing Normal University, Beijing, China; ^2^Beijing Key Laboratory of Applied Experimental Psychology, School of Psychology, Beijing Normal University, Beijing, China; ^3^Department of Psychology, College and Graduate School of Arts and Sciences, University of Virginia, Charlottesville, VA, United States; ^4^School of Kinesiology and Health, Capital University of Physical Education and Sports, Beijing, China; ^5^Department of Psychology, University of California, Berkeley, Berkeley, CA, United States; ^6^Department of Psychology, Guangzhou University, Guangzhou, China; ^7^Department of Psychology, University of Macau, Macau, China

**Keywords:** parent emotion dysregulation, harsh parenting practices, oppositional defiant disorder, child depressive symptoms, child aggressive behaviors, descriptive survey study

## Abstract

Family factors including parental emotion dysregulation and harsh parenting practices place children at high risk for malfunctioning in emotion regulation, depressive symptoms, and aggressive behaviors. This study investigated the associations among parental emotion dysregulation, harsh parenting practices (i.e., emotional abuse and corporal punishment), and child emotion regulation and child oppositional defiant disorder (ODD) symptoms and co-occurring depressive symptoms and aggressive behaviors. Participants included 239 parent–child dyads from 14 primary schools in Mainland China. All children were diagnosed with ODD. Parental emotion dysregulation, harsh parenting practices, and child emotion regulation were reported by parents; child ODD symptoms were reported by parents and teachers; child depressive symptoms were reported by children; and child aggressive behaviors were reported by teachers. Data indicated that parental emotion dysregulation was related to child ODD symptoms in the home and depressive symptoms indirectly through harsh parenting practices and child emotion regulation. Harsh parenting practices were related to child ODD symptoms in the home directly and indirectly through child emotion regulation. Moreover, emotional abuse was associated with child depressive symptoms directly and indirectly through child emotion regulation. Our findings highlighted the need for prevention and intervention targeting parent emotion dysregulation and harsh parenting practices among children with ODD.

## Introduction

For decades, research has focused on family risk factors and their strong predictive associations with child internalizing and externalizing problems. In this study we focus on two salient family risk factors linked to child Oppositional Defiant Disorder (ODD) symptoms (American Psychiatric Association [APA], 2013), depressive symptoms, and aggressive behaviors among children diagnosed with ODD: parent emotion dysregulation (Buckholdt et al., 2014) and harsh parenting practices which includes emotional abuse and corporal punishment (Gershoff, 2002; Morris and Gibson, 2012; Zhao, 2013). Moreover, we investigated the transdiagnostic role that child emotion regulation played in the effects of parent emotion dysregulation and harsh parenting practices on child ODD symptoms and co-occurring depressive symptoms and aggressive behaviors. In general, we explored the associations among parent emotion dysregulation, harsh parenting practices, child emotion regulation, and ODD symptoms, depressive symptoms and aggressive behaviors among Chinese children with ODD in the current study.

Oppositional defiant disorder refers to a recurrent and developmentally inappropriate pattern of angry/irritable, negative, defiant, disobedient, and hostile behavior toward others that causes functional impairment; and this pattern persists for at least 6 months (American Psychiatric Association [APA], 2013). ODD is one of the most commonly occurring disorders among young children (Lavigne et al., 2012) with an estimated 3.3% prevalence in the general population (American Psychiatric Association [APA], 2013). ODD is associated with malfunctioning in social, academic and occupational aspects, and family relationships over the lifespan (Burke and Romano-Verthelyi, 2018). The irritable emotion cluster of ODD includes temper tantrums and myriad forms of negative emotionality, and the defiant/headstrong behavior cluster includes being argumentative and being defiant toward adults, deliberately annoying people, and blaming others (Stringaris and Goodman, 2009; Burke et al., 2010). Children with ODD manifest a combination of emotional and behavioral impairments (American Psychiatric Association [APA], 2013) and often perform co-occurring internalizing and externalizing psychopathology symptoms such as depressive symptoms and aggressive behaviors (Steiner and Remsing, 2007; Althoff et al., 2014; Déry et al., 2017).

A number of family risk factors contribute to child psychopathology such as ODD symptoms and co-occurring depressive symptoms and aggressive behaviors (Burke et al., 2010; Suveg et al., 2011; Lavigne et al., 2012; Liu et al., 2017), among which pathways from parent emotion dysregulation and harsh parenting practices to child depressive symptoms and aggressive behaviors have been proposed (Gershoff, 2002; Morris and Gibson, 2012; Zhao, 2013; Buckholdt et al., 2014).

Researchers have highlighted the contribution of parent emotion dysregulation to child internalizing and externalizing psychopathology symptoms (Buckholdt et al., 2014). Parent emotion dysregulation was defined as having difficulties in initiating, maintaining, or modulating the occurrence, intensity, or expression of emotions (Thompson, 1994). In particular, mothers’ emotion regulation has been found associated with persistent child conduct problems from preschool to early school age (Cole et al., 2003). Fathers’ excessive expression of hostility has also been found positively associated with children’s externalizing problems such as aggression and conduct problems across time (Carrère and Bowie, 2012). However, quite contradictorily, another study indicated that parent emotion dysregulation was only associated with children’s internalizing problems such as anxiety and depression, but not with externalizing problems such as aggressive behaviors (Han and Shaffer, 2013).

Parents’ emotional dysregulation has been suggested to be associated with harsh parenting practices. Parents with emotional regulation problems may be affected to such an extent that their parent–child social interactions are impaired, which is associated with a lack of nurturing and caring support, and/or with harsh parenting practices (Cummings and Davies, 1994; Lovejoy et al., 2000; Kim and Cicchetti, 2010; Buckholdt et al., 2014) and increased risk of child maltreatment (Crandall et al., 2015). In a qualitative study of Chinese children with ODD, children reported that when parents lost their tempers, they used harsh disciplinary practices such as beating or having children’s ears boxed (Zhao, 2013). This study also suggested the potential associations between parent emotional dysregulation and harsh parenting practices in Chinese settings.

In Western settings, harsh parenting practices, particularly emotional abuse and harsh corporal punishment, are tightly associated with internalizing and externalizing problems in children (e.g., anxiety/depression, aggressive behaviors, conduct problems, and ODD) (Lansford et al., 2002; Smith and Farrington, 2004; Alink et al., 2009; Tung and Lee, 2014). In Chinese settings, harsh parenting has also been suggested as related to child psychopathology such as depressive symptoms and aggressive behaviors (Xu et al., 2009; Olson et al., 2011; Li L. et al., 2016; Tang et al., 2018). However, it should be noted that Chinese parents were reported to frequently use strict discipline with their children (Tang and Davis, 1996), and that Chinese children were more likely than those in the West to suffer from harsh parenting practices, as harsh parenting practices are considered effective parenting strategies and as good for children by many in Chinese settings where filial piety and familism values are embraced (Liao et al., 2011; Wang et al., 2014). Hence, in the Chinese cultural context, it is hard for many Chinese families to differentiate corporal punishment, which is often justified by traditional Chinese cultural values such as filial piety and viewed as good for children in the long run (Liao et al., 2011), and physical abuse that is detrimental to children. Given the cultural difference illustrated above, to what extent and how harsh parenting practices are associated with child ODD symptoms, depressive symptoms, and aggressive behaviors among Chinese children with ODD remains an issue to more thoroughly explored.

Moreover, parental emotional abuse and corporal punishment might be related to internalizing and externalizing problems in children in different ways, as mixed findings have been shown in literature. Emotional abuse has been found to not only be associated with children’s anxiety and depression, but also with non-compliance, aggressive behavior, and conduct problems (Caples and Barrera, 2006; Alink et al., 2009). Parental emotional abuse is more likely than corporal punishment to be linked to child anxiety, depressive symptoms, acute stress, and anger/hostility, and comparable to corporal punishment in predicting externalizing problems (Spinazzola et al., 2014). Parental corporal punishment has been found to be strongly associated with child aggressive, defiant, and disruptive behaviors (Shields and Cicchetti, 2001; Gershoff, 2002; Burke et al., 2004; Pardini et al., 2008). Moreover, frequent parental corporal punishment was found to be associated with the development of ODD, which includes both internalizing and externalizing symptoms, in elementary school students (Tung and Lee, 2014). However, distinct but not similar pathways were found in a recent study in Chinese children with ODD (Li L. et al., 2016), in which emotional abuse predicted child anger management but not aggressive behaviors, while parental corporal punishment predicted child aggressive behaviors but not anger management. One explanation was argued by Li L. et al. (2016) that because ODD is comprised of distinctive emotional and behavioral dimensions (Burke et al., 2010), for children with ODD, co-occurring internalizing and externalizing problems were distinctive and predicted by emotional abuse and corporal punishment separately (Li L. et al., 2016).

Last but not least, child emotional regulation has been found to play a transdiagnostic predictive role on child co-occurring internalizing and externalizing psychopathology (Aldao et al., 2016), and a transdiagnostic mediating role in the effect of family risk factors (e.g., parent emotional dysregulation and harsh parenting practices) on co-occurring internalizing and externalizing psychopathology in children (Goodman and Gotlib, 1999; Duncombe et al., 2012; Buckholdt et al., 2014; Heleniak et al., 2016). For example, child emotional regulation was found to mediate the relationship between maternal psychopathology and child psychopathology symptoms (Suveg et al., 2011). More particularly, child emotional regulation was found to mediate the effect of parent emotional dysregulation on internalizing and externalizing symptoms in a child, in a study on adolescents aged between 12 and 18 years old (Buckholdt et al., 2014). Child emotional regulation was also found to mediate the relationship between parents’ inconsistent discipline and corporal punishment and child disruptive behavior problems (Duncombe et al., 2012). Similarly, children who suffered from maltreatment were found to have more difficulties in emotional regulation compared to children who had not suffered, which were further related to higher levels of internalizing and externalizing psychopathology symptoms children, such as anxiety/depression, aggressive behaviors, and peer bullying (Shields and Cicchetti, 1997; Shields et al., 2001; Alink et al., 2009). More recently, emotional dysregulation was found to mediate the interrelationships between childhood emotional abuse, physical neglect and sexual abuse, and current adult depressive symptoms (Crow et al., 2014).

Several research questions were identified to be further explored. First, what are the pathways from parent emotional dysregulation and harsh parenting practices to child ODD symptoms, depressive symptoms, and aggressive behaviors among Chinese children with ODD? Second, in association with harsh parenting practices and child ODD symptoms, and depressive symptoms and aggressive behaviors, exactly how did emotional abuse and corporal punishment associate with child ODD symptoms, depressive symptoms, and aggressive behaviors separately? Third, to what extent did child emotional regulation mediate the detrimental effects of parental emotional dysregulation and harsh parenting practices on child ODD symptoms, depressive symptoms, and aggressive behaviors? To answer these questions, we conducted a comprehensive model depicting the associations among parental emotional dysregulation, harsh parenting practices, child emotional regulation, and child ODD symptoms and co-occurring depressive symptoms, and aggressive behaviors among Chinese children with ODD. We hypothesized that: (1) both parental emotional dysregulation and harsh parenting practices would directly predict child ODD symptoms, depressive symptoms, and aggressive behaviors, and parental emotional dysregulation would make significant indirect contributions to child ODD symptoms, depressive symptoms, and aggressive behaviors through harsh parenting practices; (2) emotional abuse and corporal punishment would predict child ODD symptoms, depressive symptoms, and aggressive behaviors through separate pathways; (3) child emotional regulation would mediate both the association between parental emotional dysregulation and child ODD symptoms, depressive symptoms, and aggressive behaviors, and the association between harsh parenting practices and child ODD symptoms, depressive symptoms, and aggressive behaviors.

## Materials and Methods

### Participants

During 2013–2014 data were collected from 14 elementary schools in the northern (Beijing), eastern (Shandong Province), and southwestern areas (Yunnan province) of Mainland China. Invitation letters that introduced our study and consent forms were sent through school psychologists to class master teachers who taught and managed classes from first grade through sixth grade in each school. In total,187 class master teachers signed and provided informed consent and were asked to nominate the children who might have ODD symptoms according to the diagnostic criteria in the *Diagnostic and Statistical Manual of Mental Disorders* (DSM-IVR; American Psychiatric Association, 2000). Of the total 7966 children, 360 were nominated from these 14 schools (4.52% of all children).

Two clinical psychologists working with school psychologists interviewed class master teachers to confirm each child’s diagnosis. Interview questions were based on the following DSM-IVR diagnostic criteria: (a) the child exhibited four or more symptoms of ODD; (b) the identified symptoms had lasted at least for 6 months or longer; and (c) significant impairments had been demonstrated across psychosocial functional domains. Only when all three of the two clinical psychologists and the one corresponding school psychologist agreed on the diagnosis of the child based on the DSM-IVR criterion; would the child be diagnosed with ODD. All uncertainties during the interview were solved based on the judgment of the two clinical psychologists.

In total, 305 children were identified with ODD and invited to participate in the present study (3.83% of all the children from these participating schools). Invitation letters and informed consent forms were sent to their parents. Later, 282 parent–child dyads signed and returned the informed consent for both themselves and their children. Out of the 282 dyads from whom consent was obtained, a total of 259 dyads entered the subsequent research procedure with completed sets of child, parent, and class teacher consent forms. Excluding other invalid questionnaires (i.e., missing data on more than half of the items in the questionnaire), the final ODD sample consisted of 239 parent–child dyads (78.36% participation rate; representing 156 classes), including 148 mother–child dyads, 71 father–child dyads, and 20 parent–child participants (missing the parent’s gender). We added the comparison between those 239 dyads who agreed and had valid data and the other 20 dyads who agreed but had invalid data. Results of one-way ANOVAs showed that those 239 dyads and the other 20 dyads did not differ in child gender, parent gender, parent education, and family monthly income (*p* > 0.05). For the scores of teacher-rated ODD symptoms of the final 239 dyads as determined by the aforementioned interviews, Cronbach’s α = 0.17.

### Survey Procedure

The Institutional Review Board of Beijing Normal University in China approved the protocol of the present study, including the informed consent procedure. We first reached 20 school psychologists to obtain their informed consent of attending the research, and 14 of them agreed. Then, these 14 school psychologists were asked to inquiry about the attending class teachers’ willingness from grade 1 to grade 6, and we obtained 187 informed consent forms from class teachers. Third, the class teachers were asked to deliver the informed consent and study introduction to the identified students. Once signed informed consent forms were obtained from parents, each child participant was asked to deliver a package, containing questionnaires for parents, to the parents who is the principal caregiver. Parents were asked to return their completed questionnaires to the class teacher within 1 week. During the school day, child participants completed questionnaires in school conference rooms or music rooms. In order to prevent the potential consequence as being treated differently by classmates, child participants were informed that they were randomly selected from their classes. As child participants were completing their questionnaires, researchers stayed with them to provide assistance if needed. In teachers’ offices, the class teachers completed questionnaires assessing child participants’ psychological attributes and behavioral performances.

As a token of appreciation for their participation, each of the teachers received $8 and each of the 305 children who met the ODD criteria and their parents were provided a treatment opportunity with psychiatrists in Anding Hospital and psychological counselors and family therapists at the Center of Family Study and Therapy at the Institute of Developmental in Beijing Normal University.

### Instruments

#### Parents’ Emotion Dysregulation (Parent Reported)

We used the Difficulties in Emotion Regulation Scale (DERS; Gratz and Roemer, 2004) to assess parental emotion dysregulation. It has been translated to a Chinese version and used among Chinese population (Han et al., 2015; Lin et al., 2016). The DERS contains 36 items (e.g., “I experience my emotions as overwhelming and out of control;” “I have difficulty making sense out of my feelings”) and 6 dimensions (Awareness, Non-acceptance, Goals, Impulsive, Strategies, and Clarity). The Awareness dimension was excluded for its weak convergence with the other dimensions and its weak factor loading (Bardeen et al., 2012). Parents’ self-reported responses on the DERS were rated on a five-point scale (1 = *almost never*, 5 = *almost always*). Summed scores served as an indicator of parents’ emotional dysregulation (Cronbach’s α = 0.91 in the current study). Scores ranged from 29 to 155, with higher scores indicating greater emotional dysregulation.

#### Children’s Emotion Regulation (Parent Reported)

Parents’ evaluations of children’s emotional regulation were assessed using the Emotion Regulation Checklist (ERC; Shields and Cicchetti, 1997; Shields et al., 2001). The ERC has been translated into a Chinese version and showed good reliability in the Chinese sample (Han et al., 2015; Lin et al., 2016). The ERC contains 23 items indicating how frequently a child manifests affective behavior that is rated on four-point scales (1 = *never*, 4 = *always*). The checklist is divided into two subscales: Emotion Regulation and Lability/Negativity. The former assesses appropriate emotional expression, empathy, and emotional self-awareness [e.g., “Can modulate excitement in emotionally arousing situations” (reversed)] and the later measures inflexibility, lability, and dysregulated negative effects (e.g., “Exhibits wide mood swings”). Summed scores served as an indicator of child emotional regulation (Cronbach’s α = 0.83 in the current study). Scores ranged from 23 to 92, with higher scores indicating severe emotional regulation problems.

#### Parental Emotional Abuse (Parent Reported)

The Emotional Abuse subscale of Childhood Trauma Questionnaire (CTQ; Bernstein and Fink, 1998) was used to measure parental emotional abuse. The Emotional Abuse subscale was administered in a Chinese version translated and revised by Zhao et al. (2005). The five items related to emotional abuse (e.g., “People in your family called him/her things like stupid, lazy, or ugly”) were reported by parents using five-point scales (1 = *never*, 5 = *very often*). Scores of the five items were summed to create composite scores (Cronbach’s α for the Emotional Abuse subscale was 0.87 in the current study). Potential scores ranged from 5 to 25, with higher scores indicating higher levels of emotional abuse toward children.

#### Parental Corporal Punishment (Parent Reported)

We selected seven items from the Conflict Tactics Scale (CTS; Straus, 1979; Straus and Mickey, 2012) to assess parental harsh discipline. The selected seven items were translated into a Chinese version and showed good reliability in a previous study on migrant children with ODD symptoms in China (Li Y. et al., 2016). Parents were asked to report how often he or she displayed physical aggression toward their children (e.g., “Pushed, grabbed, or shoved child;” “Hit or tried to hit with something”) on a seven-point scale (0 = *never*, 6 = *almost every day*). Summed scores of these seven items act as an indicator of parental harsh discipline (Cronbach’s α of the seven items were 0.93 in the current study). Potential scores ranged from 0 to 66, with higher scores indicating higher levels of punishment.

#### Parent-Rated ODD Symptoms

Children’s ODD symptoms in the family context were measured by eight items (e.g., “often deliberately annoys people;” “often argues with adults”) based on diagnostic criteria listed in DSM-IVR (American Psychiatric Association, 2000). A previous study suggested good internal consistency for the eight items (Cronbach’s α = 0.93; Lindhiem et al., 2015). A Chinese version of the DSM-IVR criteria of ODD (Huang et al., 2006) was used in the study. Each parent rated their child’s ODD symptoms on scales (0 = *no*, 1 = *yes*). The summed scores of eight items were calculated and used as the indicator of ODD symptoms in the home (Cronbach’s α = 0.85 in the current study). Potential scores ranged from 0 to 8, with higher scores indicating more severe ODD symptoms.

#### Teacher-Rated ODD Symptoms

Children’s ODD symptoms in the school context were reported by teachers working with clinical psychologists, using the eight items (e.g., “often deliberately annoys people;” “often argues with adults”) from the diagnostic criteria listed in DSM-IVR (American Psychiatric Association, 2000). A Chinese version of the criteria (Huang et al., 2006) was used in the study. Each teacher rated the children’s ODD symptoms on scales (0 = *no*, 1 = *yes*). The summed scores of eight items were used as the indicator of ODD symptoms in school (Cronbach’s α = 0.17 in the current study). The relatively low Cronbach’s α might be due to the skewed distribution of scores as all children were identified with ODD (i.e., teacher-rated ODD symptoms ≥4), and further might be due to the underlying multi-dimensionality and low correlations between items (Graham, 2006; Agbo, 2010). In the case of skewed scores, the McDonald’s ω and Greatest Lower Bound (GLB) were calculated as alternatives using JASP (McDonald’s ω = 0.22 and GLB = 0.47 in the current study; Dunn et al., 2014; Trizano-Hermosilla and Alvarado, 2016; Goss-Sampson, 2019).

#### Children’s Depressive Symptoms (Child Reported)

Children’s reports of depressive symptoms were assessed using the Center for Epidemiological Studies Depression Scale for Children (CES-DC) (Fendrich et al., 1990). The scale was translated to a Chinese version and showed good reliability in a previous study on Chinese children with ODD symptoms (Lin et al., 2014). This measure contains 20 items rated on a four-point scale (0 = *not at all*, 1 = *a little*, 2 = *some*, 3 = *a lot*). The following two statements are examples of the items: “I was bothered by things that usually don’t bother me” and “My appetite was poor.” Higher summed scores serve as a severe indicator of children’s depressive symptoms (Cronbach’s α = 0.86 in the current study). Potential scores range from 0 to 60, with higher scores indicating higher levels of depressive symptoms.

#### Children’s Aggressive Behaviors (Teacher Reported)

Children’s aggressive behavior toward peers were assessed by the aggression subscale of the Child Behavior Scale (CBS; Ladd and Profilet, 1996). The aggression subscale was translated to a Chinese version and proved to have good reliability in a previous study on the Chinese population (Lin et al., 2014). Class teachers evaluated children’s aggressive behavior toward their peers using five-point scales (1 = *never*, 5 = *always*) on seven items (e.g., “This child pushes or shoves other children”). Scores of these seven items were summed to create a composite score (Cronbach’s α = 0.94 in the current study). Scores ranged from 5 to 35, with higher scores indicating more aggressive behaviors.

### Data Analyses

First, we used SPSS 20.0 to analyze descriptive characteristics of our sample. Next, we computed Pearson correlations to examine associations between variables. Finally, structural models were examined to illuminate how family risk factors were associated with psychopathology symptoms for children with ODD *via* Mplus version 8.2 (Muthén and Muthén, (1998-2017)). Bias-corrected 95% confidence intervals for path estimates were generated *via* bootstrapping with 5000 iterations (Preacher and Hayes, 2008).

## Results

### Characteristics of Sample

Child participants included 171 boys (71.55%) and 68 girls (28.45%) aged between 6 and 12 years (*M* = 9.60, *SD* = 1.59). Among these children, 190 (79.4%) children were identified as the only child in the family, 44 (18.4%) had one sibling, and four (1.7%) had two siblings (one missing data). Fathers’ ages ranged from 25 to 54 years old (*M* = 38.40, *SD* = 5.03) and mothers’ ages ranged from 26 to 53 years old (*M* = 36.63, *SD* = 4.17). For education, 39.4% fathers and 36.2% mothers had bachelor degrees or a higher education, 21.6% fathers and 21.0% mothers had a junior college diploma, 18.2% fathers and 19.7% mothers had a high school diploma or technical secondary school diploma, and the remaining 20.8% fathers and 23.1% mothers had a middle school diploma or lower. For family income, 25.6% of the families had a monthly income >10,000 CNY (6.60 CNY ≈ 1 USD), 31.2% had 5000–10,000 CNY, 35.3% had 2000–5000 CNY, and 7.9% had <2000 CNY. The range of teacher-reported ODD symptoms ranged from 4 to 8 (*M* = 5.41, *SD* = 1.36), and for parent-reported symptoms ranged from 0 to 8 (*M* = 2.64, *SD* = 2.52).

### Correlations Between Variables

Correlations between variables are presented in Table 1. As expected, parental emotional dysregulation, emotional abuse, corporal punishment, child emotional regulation, child depressive symptoms, and parent-rated ODD symptoms were significantly intercorrelated. Corporal punishment, parent-rated ODD symptoms, teacher-rated ODD symptoms, and depressive symptoms were significantly correlated with aggressive behavior in the child. Parent-rated ODD symptoms and teacher-rated symptoms were significantly correlated. Moreover, child age was significantly correlated with teacher-rated ODD symptoms, and paternal and maternal age were significantly correlated to teacher-rated ODD symptoms and aggressive behaviors, which were controlled in further analyses.

**TABLE 1:** Correlation coefficients of variables.

	***M* (*SD*)**	**1**	**2**	**3**	**4**	**5**	**6**	**7**	**8**
1. DERS	76.53 (14.17)	1.00							
2. Emotional abuse	8.13 (3.43)	0.52^∗∗∗^	1.00						
3. Corporal punishment	10.04 (8.68)	0.43^∗∗∗^	0.68^∗∗∗^	1.00					
4. ERC	47.80 (8.31)	0.45^∗∗∗^	0.62^∗∗∗^	0.55^∗∗∗^	1.00				
5. Parent-rated ODD symptoms	2.64 (2.52)	0.28^∗∗∗^	0.61^∗∗∗^	0.55^∗∗∗^	0.71^∗∗∗^	1.00			
6. Teacher-rated ODD symptoms	5.41 (1.36)	–0.12	0.03	0.08	0.11	0.20^∗∗^	1.00		
7. Depressive symptoms	16.07 (10.31)	0.21^∗∗^	0.40^∗∗∗^	0.32^∗∗∗^	0.38^∗∗∗^	0.40^∗∗∗^	0.12	1.00	
8. Aggressive behaviors	17.68 (7.68)	–0.07	0.10	0.16^∗^	0.13	0.16^∗^	0.48^∗∗∗^	0.19^∗∗^	1.00
9. Child age	0.28 (0.45)	–0.003	0.05	–0.02	–0.04	–0.03	0.14^∗^	0.003	0.05
10. Paternal age	38.40 (5.03)	–0.11	0.02	–0.02	0.07	0.10	0.22^∗∗^	0.05	0.25^∗∗∗^
11. Maternal age	36.63 (4.17)	–0.12	0.001	–0.06	–0.03	0.02	0.17^∗∗^	–0.03	0.26^∗∗∗^

*^∗^*p* < 0.05, ^∗∗^*p* < 0.01, ^∗∗∗^*p* < 0.001. DERS, Difficulties in Emotion Regulation Scale; ERC, Emotion Regulation Checklist; ODD, Oppositional Defiant Disorder.*

Additionally, one-way ANOVAs were conducted to analyze whether parental emotional dysregulation, emotional abuse, corporal punishment, child emotional regulation, parent-rated ODD symptoms, teacher-rated ODD symptoms, child depressive symptoms, and child aggressive behaviors differed between child gender, parent gender, parent education (including paternal and maternal education), and family monthly income. Results are presented in Table 2. Child gender, parent education, and family monthly income were controlled in following analyses.

**TABLE 2:** ANOVA analyses.

	**Child gender**	**Parent gender**	**Paternal education**	**Maternal education**	**Family monthly income**
DERS	*p* > 0.05	*p* > 0.05	*F*(5,204) = 4.09, *p* < 0.05	*F*(5,203) = 3.06, *p* < 0.05	*F*(4,204) = 5.10, *p* < 0.05
Emotional abuse	*p* > 0.05	*p* > 0.05	*F*(5,219) = 3.22, *p* < 0.05	*p* > 0.05	*F*(4,220) = 3.08, *p* < 0.05
Corporal punishment	*p* > 0.05	*p* > 0.05	*F*(5,228) = 3.25, *p* < 0.05	*p* > 0.05	*F*(4,228) = 3.27, *p* < 0.05
ERC	*F*(1,218) = 4.72, *p* < 0.05	*p* > 0.05	*F*(5,212) = 4.41, *p* < 0.05	*p* > 0.05	*p* > 0.05
Parent-rated ODD symptoms	*F*(1,231) = 5.99, *p* < 0.05	*p* > 0.05	*F*(5,225) = 6.74, *p* < 0.05	*F*(5,224) = 2.71, *p* < 0.05	*F*(4,226) = 4.57, *p* < 0.05
Teacher-rated ODD symptoms	*F*(1,236) = 17.39, *p* < 0.05	*p* > 0.05	*p* > 0.05	*p* > 0.05	*p* > 0.05
Depressive symptoms	*F*(1,213) = 6.06, *p* < 0.05	*p* > 0.05	*F*(5,208) = 2.82, *p* < 0.05	*p* > 0.05	*F*(4,208) = 2.69, *p* < 0.05
Aggressive behaviors	*F*(1,231) = 25.73, *p* < 0.05	*p* > 0.05	*F*(5,225) = 4.26, *p* < 0.05	*F*(5,224) = 2.75, *p* < 0.05	*F*(4,225) = 4.28, *p* < 0.05

### Path Analyses: Mediation Model

**FIGURE 1:**
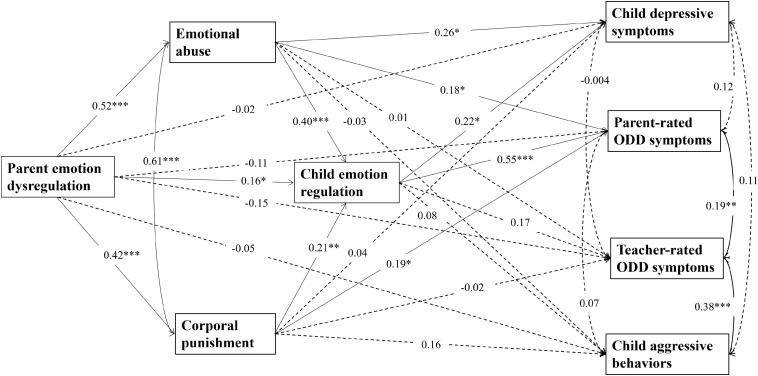
Structural equation model. Dotted arrows represent no significant results while solid arrows mean significant results. Standardized path coefficients are on the single-headed arrows; correlation appears on curved double-headed arrow. Children’s age and gender, paternal age, maternal age, paternal education, maternal education, and family monthly income are controlled in the model (not shown). ^∗^*p* < 0.05, ^∗∗^*p* < 0.01, and ^∗∗∗^*p* < 0.001.

Results of multiple tests for mediation effects are shown in Table 3. The structural model (shown in Figure 1) indicated a good model fit: χ^2^(28) = 42.72, *p* = 0.04; RMSEA = 0.05; CFI = 0.98; TLI = 0.94; SRMR = 0.05. Parental emotional dysregulation had no direct effects on child parent-rated ODD symptoms and teacher-rated ODD symptoms, depressive symptoms, or aggressive behaviors. However, parental emotional dysregulation had a significant indirect effect on child parent-rated ODD symptoms through emotional abuse, corporal punishment, and child emotional regulation separately, and through emotional abuse and child emotional regulation in sequence, and corporal punishment and child emotional regulation in sequence. Associations between parental emotional dysregulation and child depressive symptoms were mediated by emotional abuse, and by emotional abuse and child emotional regulation in sequence. However, there was no significant direct or indirect link between parental emotional dysregulation and child teacher-rated ODD symptoms or aggressive behaviors.

**TABLE 3:** Mediation testing in the structural model.

**Paths**	**β**	**95% CI**	***p***
**Direct effect**			
Emotional abuse – parent-rated ODD symptoms	0.18	[0.03, 0.34]	0.02
Corporal punishment – parent-rated ODD symptoms	0.19	[0.03, 0.35]	0.02
Emotional abuse – depressive symptoms	0.26	[0.04, 0.51]	0.03
Parent emotion dysregulation – child emotion regulation	0.16	[0.04, 0.29]	0.01
**Indirect effect**			
Parent emotion dysregulation – emotional abuse – parent-rated ODD symptoms	0.09	[0.02, 0.19]	0.03
Parent emotion dysregulation – corporal punishment – parent-rated ODD symptoms	0.08	[0.02, 0.16]	0.03
Parent emotion dysregulation – child emotion regulation – parent-rated ODD symptoms	0.09	[0.02, 0.17]	0.02
Parent emotion dysregulation – emotional abuse – child emotion regulation – parent-rated ODD symptoms	0.11	[0.07, 0.18]	<0.001
Parent emotion dysregulation – corporal punishment – child emotion regulation – parent-rated ODD symptoms	0.05	[0.02, 0.10]	0.01
Parent emotion dysregulation – emotional abuse – teacher-rated ODD symptoms	0.01	[−0.10, 0.12]	0.91
Parent emotion dysregulation – corporal punishment – teacher-rated ODD symptoms	–0.01	[−0.09, 0.07]	0.81
Parent emotion dysregulation – child emotion regulation – teacher-rated ODD symptoms	0.03	[0.00, 0.08]	0.16
Parent emotion dysregulation – emotional abuse – child emotion regulation – teacher-rated ODD symptoms	0.04	[0.001, 0.04]	0.10
Parent emotion dysregulation – corporal punishment – child emotion regulation – teacher-rated ODD symptoms	0.02	[0.001, 0.09]	0.13
Parent emotion dysregulation – emotional abuse – depressive symptoms	0.14	[0.02, 0.28]	0.03
Parent emotion dysregulation – child emotion regulation – depressive symptoms	0.04	[0.004, 0.09]	0.11
Parent emotion dysregulation – emotional abuse – child emotion regulation – depressive symptoms	0.05	[0.02, 0.10]	0.02
Parent emotion dysregulation – corporal punishment – child emotion regulation – depressive symptoms	0.02	[0.004, 0.05]	0.08
Parent emotion dysregulation – corporal punishment – depressive symptoms	0.02	[−0.07, 0.11]	0.67
Parent emotion dysregulation – emotional abuse – child emotion regulation	0.21	[0.12, 0.31]	<0.001
Parent emotion dysregulation – corporal punishment – child emotion regulation	0.09	[0.04, 0.16]	0.003
Parent emotion dysregulation – child emotion regulation – aggressive behaviors	0.01	[−0.02, 0.06]	0.48
Parent emotion dysregulation – emotional abuse – aggressive behaviors	–0.02	[−0.14, 0.11]	0.82
Parent emotion dysregulation – corporal punishment – aggressive behaviors	0.07	[−0.01, 0.15]	0.10
Parent emotion dysregulation – emotional abuse – child emotion regulation – aggressive behaviors	0.02	[−0.03, 0.06]	0.42
Parent emotion dysregulation – corporal punishment – child emotion regulation – aggressive behaviors	0.01	[−0.01, 0.03]	0.45
Emotional abuse – child emotion regulation – parent-rated ODD symptoms	0.22	[0.14, 0.32]	<0.001
Emotional abuse – child emotion regulation – teacher-rated ODD symptoms	0.07	[0.00, 0.16]	0.09
Emotional abuse – child emotion regulation – depressive symptoms	0.09	[0.03, 0.17]	0.02
Emotional abuse – child emotion regulation – aggressive behaviors	0.03	[−0.05, 0.12]	0.42
Corporal punishment – child emotion regulation – parent-rated ODD symptoms	0.12	[0.05, 0.20]	0.003
Corporal punishment – child emotion regulation – teacher-rated ODD symptoms	0.04	[0.001, 0.09]	0.11
Corporal punishment – child emotion regulation – depressive symptoms	0.05	[0.01, 0.12]	0.06
Corporal punishment – child emotion regulation – aggressive behaviors	0.02	[−0.02, 0.07]	0.45

*Significant direct effect and all indirect effects are presented in this table.*

In addition, emotional abuse was related to child parent-rated ODD symptoms directly and indirectly through child emotional regulation. Corporal punishment was linked to child parent-rated ODD symptoms directly and indirectly through child emotional regulation. Similarly, emotional abuse correlated to child depressive symptoms directly and indirectly through child emotional regulation. However, the paths from emotional abuse, corporal punishment, and child emotional regulation to child teacher-rated ODD symptoms and to aggressive behaviors were not significant. Importantly, parental emotional dysregulation was linked to child emotional regulation directly and indirectly *via* emotional abuse and corporal punishment.

## Discussion

The current study examined associations between family risk factors and child ODD symptoms, and co-occurring depressive symptoms, and aggressive behaviors among families living in Mainland China. Our findings advanced knowledge of how these family risk factors were associated with children’s emotional and behavioral maladjustment through influences on child emotional regulation. Consistent with our hypotheses, parental emotional dysregulation was related to child ODD symptoms in the home and depressive symptoms indirectly through harsh parenting practices and child emotional regulation. Also, harsh parenting practices had both direct and indirect effects on child ODD symptoms in the home and depressive symptoms through child emotional regulation. Moreover, emotional dysregulation may be transmitted from parents to children directly and indirectly through harsh parenting practices. These findings highlight that interventions that are aimed at improving parents’ abilities in emotional regulation, parenting practices, and children’s skills in emotional regulation might be helpful in mitigating ODD symptoms and co-occurring depressive symptoms for children diagnosed with ODD.

As expected, parental emotional dysregulation combined with harsh parenting practices contributed to children’s ODD symptoms in the home as well as to depressive symptoms. These results extended previous research on the associations between family risk factors and child ODD symptoms and co-occurring depressive symptoms by including both parental emotional dysregulation and harsh parenting practices in a comprehensive model. Consistent with previous studies, our findings indicate that parents with difficulties in emotional regulation were more likely to adopt harsh parenting practices such as emotional abuse and corporal punishment when interacting with children (e.g., Lovejoy et al., 2000). These family risk factors might place children in stressful and insecure family contexts, which has been shown to impair children’s emotional regulation skills (Morris et al., 2007) and increase symptoms of ODD and depression (Burke et al., 2004; Morris and Gibson, 2012; Han and Shaffer, 2013).

In line with prior studies conducted in Western countries (Alink et al., 2009; Crow et al., 2014; Tung and Lee, 2014), we found that emotional abuse and corporal punishment were also related to child parent-rated ODD symptoms and self-reported depressive symptoms in a Chinese cultural context. Exposure to emotional abuse and corporal punishment differed in their associations with child ODD symptoms and depressive symptoms. Both direct and indirect association between emotional abuse and child depressive symptoms were found, with the mediation through child emotional regulation. No direct associations between corporal punishment and child depressive symptoms were found, suggesting that corporal punishment was more likely to be directly linked to ODD, which consists of both affective and behavioral symptoms (Burke et al., 2010), than to depressive symptoms. In line with a prior study in Chinese children with ODD (Li L. et al., 2016), corporal punishment could not directly predict internalizing problems (e.g., anger management and depressive symptoms), supporting the indication of the distinctive affective and behavioral dimensions of ODD (Burke et al., 2010). An indirect association between corporal punishment and child depressive symptoms was found through the mediation of child emotional regulation, highlighting the protective role of child emotional regulation in the onset of depressive symptoms for children with ODD who suffer corporal punishment.

One key finding of this study was that child emotional regulation mediated relationships between family risk factors and child ODD symptoms in the home and in depressive symptoms. Specifically, results indicate that children whose parents reported higher levels of emotional dysregulation were likely to have more difficulties in emotional regulation as well, which might exacerbate symptoms of ODD. Additionally, children who were exposed to higher levels of emotional abuse had more emotional regulation difficulties than others, which were further related to elevated levels of ODD and depressive symptoms. Similarly, frequent and severe corporal punishment was associated with greater difficulties in emotional regulation, which, in turn, were related to higher levels of ODD symptoms. These findings replicated previous research suggesting the mediating effect of child emotional regulation on the relationship between harsh parenting practices and child psychopathology symptoms (Alink et al., 2009; Crow et al., 2014), and extended the literature by examining the mechanism in a sample of Chinese children with ODD. Furthermore, these results emphasized that child emotional regulation is not only closely related to a wide range of psychopathologies (Crow et al., 2014; Graziano and Garcia, 2016), but is also an important mechanism through which family risk factors, including family emotional dysregulation and harsh parenting practices, might impair child mental health. Therefore, interventions for child psychopathology symptoms such as ODD symptoms and depressive symptoms should target both family risk factors and child emotional regulation.

It is worth noting that children’s emotional regulation is closely associated with parent’s emotional dysregulation directly and indirectly. According to Morris et al. (2007), one crucial way in which children learn about emotional regulation is by observing how parents display and manage emotions; subsequently, children might imitate and apply these observed strategies to manage their own emotions. Findings in this study offered support for the modeling hypothesis and further advanced our understanding of the potential mechanisms underlying the transmission of emotion regulation. Results indicated that parental emotional dysregulation might affect how parents interact with their children, which in turn, may impair child emotional regulation. More specifically, parents who had difficulties in emotion regulation were more likely to utilize harsh parenting practices such as emotional abuse and corporal punishment, and these harsh parenting practices were predominant risk factors for emotional regulation impairments in children.

However, results were inconsistent with previous studies that demonstrated the adverse effects of family risk factors such as corporal punishment on aggressive behaviors (Gershoff, 2002; Lansford et al., 2002) and showed mixed findings on ODD symptoms, as no such effect was found on teacher-rated ODD symptoms (i.e., ODD symptoms in the school). The first possible explanation was that this study is different from some prior studies that recruited children who were victims of childhood maltreatment; children in the current study did not suffer from severe physical abuse, which might contribute to different results. The second possible explanation was regarding different cultural contexts. As corporal punishment is viewed by many in Chinese settings as an effective parenting practice (Liao et al., 2011; Wang et al., 2014), the effect of corporal punishment on child aggressive behavior was not as strong as suggested in prior studies (Gershoff, 2002; Lansford et al., 2002). The third possible explanation was that as child aggressive behavior and teacher-rated ODD symptoms were reported by teachers in school settings, while family risk factors such as corporal punishment was rated by parents in the home environment, situation-specific factors such as children’s different functioning across different settings (Achenbach et al., 1987; Van der Oord et al., 2006) might lead to the insignificance of the pathway from family risk factors to child aggressive behaviors or teacher-rated ODD symptoms. Specifically, contradictory to a prior study in Chinese children with ODD (Li L. et al., 2016), which showed a moderate significant positive correlation (*r* = 0.20) between parental corporal punishment and child aggressive behaviors and a significant predictive pathway from corporal punishment to aggressive behaviors (β = 0.16), the current study found a moderate significant positive correlation (*r* = 0.16) but not such a significant pathway (β = 0.16). The seemingly contradicting findings of the study by Li L. et al. (2016) and the current study might be explained by the low to moderate cross-informant agreement, as the coefficients of the pathways of the prior and current studies were the same but the significance results were different.

It should be noted that the teacher–caregiver cross-informant agreement on child psychopathology including ODD has often been reported low to moderate, which might be due to factors such as different performances of children across settings (i.e., situation-specific functioning of children; Achenbach et al., 1987; Van der Oord et al., 2006; Liu et al., 2011; Gomez, 2014; Lavigne et al., 2015). The correlation between teacher-rated and parent-rated ODD symptoms in the current study was relatively low as well, comparable to the above-mentioned studies. As results showed, the current model indicated the mechanism of how family risk factors impact on child ODD symptoms and on co-occurring depressive symptoms in the family context rather than in the school context, suggesting the importance of considering informant disagreement across contexts in research on child ODD. Future studies are needed to further investigate how this teacher–parent disagreement might lead to different pathways from family and school risk factors to child ODD symptoms and other psychopathological problems.

Several limitations of the present study should be addressed. First, although the cross-sectional design of this study provided some important information about associations between family and child risk factors and child ODD symptoms, depressive symptoms, and aggressive behaviors in Chinese children with ODD, no causal relationships could be assumed. Therefore, longitudinal research is needed to test the longitudinal consequences of family risk factors on child development and the mediating role of child emotion regulation. Second, child ODD symptoms that were rated by their parents might be specific to the home setting. In contrast, teacher-rated children’s ODD symptoms and aggressive behaviors, which were not significantly associated with all the other variables in the comprehensive model, suggesting a potential different mechanism in the school settings. Third, parents reported only the scores of the eight dichotomous items based on the DSM-IVR diagnostic criteria of ODD (American Psychiatric Association, 2000) but no other more comprehensive procedure was conducted. Thus, the parent-rated ODD symptoms cannot be used for clinical diagnosis, as it represented only the ODD symptoms reported by parents. Finally, the measurement of emotional abuse and corporal punishment was solely based on parents’ self-reports. It is possible that these harsh parenting practices are underestimated. Hence, we recommend future research using multi-informant approaches and investigating cross-setting validity, considering the cross-informant agreement specifically.

Despite these limitations, our study had several strengths. First, we included both parental emotional dysregulation and harsh parenting practices as family risk factors and examined the nature of their associations with children’s ODD symptoms and co-occurring depressive symptoms and aggressive behaviors, enhancing our understanding of child psychopathology symptoms in a broader family context. Second, child emotional regulation was included in the model and was examined as a transdiagnostic mediator, which may help explain the underlying mechanisms of the association between family factors and internalizing and externalizing psychopathology in children. Third, our focus on children who were diagnosed with ODD has important implications for the prevention and treatment of ODD and co-occurring depressive symptoms. Finally, whereas most previous studies have been conducted in Western countries, this study generalized and extended prior findings in a different cultural context, Mainland China.

## Data Availability

The raw data supporting the conclusions of this manuscript will be made available by the authors, without undue reservation, to any qualified researcher.

## Ethics Statement

We declare that this research was approved by the IRB in Beijing Normal University (in China), and all the participants agreed to attend the research by signing the informed content.

## Author Contributions

XL and YL completed the majority of this manuscript. SX did the data analysis and wrote the discussion section. WD, QZ, HD, and PC provided the idea regarding the entire manuscript and gave several revision and editions.

## Conflict of Interest Statement

The authors declare that the research was conducted in the absence of any commercial or financial relationships that could be construed as a potential conflict of interest.

## References

[B1] Achenbach T. M. McConaughy S. H. Howell C. T. (1987). Child/adolescent behavioral and emotional problems: implications of cross-informant correlations for situational specificity. *Psychol. Bull.* 101 213–232. 10.1037/0033-2909.101.2.213 3562706

[B2] Agbo A. A. (2010). Cronbach’s alpha: review of limitations and associated recommendations. *J. Psychol. Afr.* 20 233–239. 10.1080/14330237.2010.10820371

[B3] Aldao A. Gee D. G. De Los Reyes A. Seager I. (2016). Emotion regulation as a transdiagnostic factor in the development of internalizing and externalizing psychopathology: current and future directions. *Dev. Psychopathol.* 28 927–946. 10.1017/S0954579416000638 27739387

[B4] Alink L. R. Cicchetti D. Kim J. Rogosch F. A. (2009). Mediating and moderating processes in the relation between maltreatment and psychopathology: mother-child relationship quality and emotion regulation. *J. Abnorm. Child Psychol.* 37 831–843. 10.1007/s10802-009-9314-4 19301118 PMC2708329

[B5] Althoff R. R. Kuny-Slock A. V. Verhulst F. C. Hudziak J. J. van der Ende J. (2014). Classes of oppositional-defiant behavior: concurrent and predictive validity. *J. Child Psychol. Psychiatry* 55 1162–1171. 10.1111/jcpp.12233 24673629 PMC4159429

[B6] American Psychiatric Association (2000). *Diagnostic and Statistical Manual of Mental Disorders*, 4th Edn Washington, DC: American Psychiatric Association.

[B7] American Psychiatric Association [APA] (2013). *Diagnostic and Statistical Manual of Mental Disorders*, 5th Edn Washington, DC: American Psychiatric Association.

[B8] Bardeen J. R. Fergus T. A. Orcutt H. K. (2012). An examination of the latent structure of the difficulties in emotion regulation scale. *J.. Psychopathol. Behav. Assess.* 34 382–392. 10.1007/s10862-012-9280-y

[B9] Bernstein D. P. Fink L. (1998). *Childhood Trauma Questionnaire: A Retrospective Self-Report Manual.* San Antonio, TX: Psychological Corporation.

[B10] Buckholdt K. E. Parra G. R. Jobe-Shields L. (2014). Intergenerational transmission of emotion dysregulation through parental invalidation of emotions: implications for adolescent internalizing and externalizing behaviors. *J. Child Fam. Stud.* 23 324–332. 10.1007/s10826-013-9768-4 24855329 PMC4024378

[B11] Burke J. D. Hipwell A. E. Loeber R. (2010). Dimensions of oppositional defiant disorder as predictors of depression and conduct disorder in preadolescent girls. *J. Am. Acad. Child Adoles. Psychiatry* 49 484–492. 10.1016/j.jaac.2010.01.016 20431468 PMC2880833

[B12] Burke J. D. Loeber R. Birmaher B. (2004). Oppositional defiant disorder and conduct disorder: a review of the past 10 years, part II. *Focus* 41 1275–1293. 10.1176/foc.2.4.558 12410070

[B13] Burke J. D. Romano-Verthelyi A. M. (2018). “Oppositional defiant disorder,” in *Developmental Pathways to Disruptive, Impulse-Control and Conduct Disorders*, ed. Martel M. M., (Cambridge, MA: Academic Press.), 21–52. 10.1016/C2016-0-00396-0

[B14] Caples H. S. Barrera M. Jr. (2006). Conflict, support and coping as mediators of the relation between degrading parenting and adolescent adjustment. *J. Youth Adoles.* 35 599–611. 10.1007/s10964-006-9057-2

[B15] Carrère S. Bowie B. H. (2012). Like parent, like child: parent and child emotion dysregulation. *Arch. Psychiatr. Nurs.* 26 23–30. 10.1016/j.apnu.2011.12.008 22633588

[B16] Cole P. M. Teti L. O. Zahn-Waxler C. (2003). Mutual emotion regulation and the stability of conduct problems between preschool and early school age. *Dev. Psychopathol.* 15 1–18. 10.1017/S0954579403000014 12848432

[B17] Crandall A. Deater-Deckard K. Riley A. W. (2015). Maternal emotion and cognitive control capacities and parenting: a conceptual framework. *Dev. Rev.* 36 105–126. 10.1016/j.dr.2015.01.004 26028796 PMC4445866

[B18] Crow T. Cross D. Powers A. Bradley B. (2014). Emotion dysregulation as a mediator between childhood emotional abuse and current depression in a low-income African-American sample. *Child Abuse Neglect* 38 1590–1598. 10.1016/j.chiabu.2014.05.015 25035171 PMC4254147

[B19] Cummings E. M. Davies P. T. (1994). Maternal depression and child development. *J. Child Psychol. Psychiatry* 35 73–112. 10.1111/j.1469-7610.1994.tb01133.x 8163630

[B20] Déry M. Lapalme M. Jagiellowicz J. Poirier M. Temcheff C. Toupin J. (2017). Predicting depression and anxiety from oppositional defiant disorder symptoms in elementary school-age girls and boys with conduct problems. *Child Psychiatry Hum. Dev.* 48 53–62. 10.1007/s10578-016-0652-5 27209374

[B21] Duncombe M. E. Havighurst S. S. Holland K. A. Frankling E. J. (2012). The contribution of parenting practices and parent emotion factors in children at risk for disruptive behavior disorders. *Child Psychiatry Hum. Dev.* 43 715–733. 10.1007/s10578-012-0290-5 22392414

[B22] Dunn T. J. Baguley T. Brunsden V. (2014). From alpha to omega: a practical solution to the pervasive problem of internal consistency estimation. *Br. J. Psychol.* 105 399–412. 10.1111/bjop.12046 24844115

[B23] Fendrich M. Weissman M. M. Warner V. (1990). Screening for depressive disorder in children and adolescents: validating the center for epidemiologic studies depression scale for children. *Am. J. Epidemiol.* 131 538–551. 2301363 10.1093/oxfordjournals.aje.a115529

[B24] Gershoff E. T. (2002). Corporal punishment by parents and associated child behaviors and experiences: a meta-analytic and theoretical review. *Psychol. Bull.* 128 539–579. 10.1037/0033-2909.128.4.539 12081081

[B25] Gomez R. (2014). Malaysian parent and teacher ratings of the oppositional defiant disorder symptoms: measurement invariance and parent–teacher agreement. *Asian J. Psychiatry* 11 35–38. 10.1016/j.ajp.2014.05.002 25453694

[B26] Goodman S. H. Gotlib I. H. (1999). Risk for psychopathology in the children of depressed mothers: a developmental model for understanding mechanisms of transmission. *Psychol. Rev.* 106 458–490. 10.1037/0033-295X.106.3.458 10467895

[B27] Goss-Sampson M. A. (2019). *Statistical Analysis in JASP 0.10.2: A Guide for Students.* Available at: https://static.jasp-stats.org/Statistical%20Analysis%20in%20JASP%20-%20A%20Students%20Guide%20v0.10.2.pdf (accessed July 30, 2019).

[B28] Graham J. M. (2006). Congeneric and (essentially) tau-equivalent estimates of score reliability: what they are and how to use them. *Educ. Psychol. Meas.* 66 930–944. 10.1177/0013164406288165

[B29] Gratz K. L. Roemer L. (2004). Multidimensional assessment of emotion regulation and dysregulation: development, factor structure, and initial validation of the difficulties in emotion regulation scale. *J. Psychopathol. Behav. Assess.* 26 41–54. 10.1007/s10862-008-9102-4

[B30] Graziano P. A. Garcia A. (2016). Attention-deficit hyperactivity disorder and children’s emotion dysregulation: a meta-analysis. *Clin. Psychol. Rev.* 46 106–123. 10.1016/j.cpr.2016.04.011 27180913

[B31] Han Z. R. Qian J. Gao M. Dong J. (2015). Emotion socialization mechanisms linking Chinese fathers’, mothers’, and dhildren’s emotion regulation: a moderated mediation model. *J. Child Fam. Stud.* 24 3570–3579. 10.1007/s10826-015-0158-y

[B32] Han Z. R. Shaffer A. (2013). The relation of parental emotion dysregulation to children’s psychopathology symptoms: the moderating role of child emotion dysregulation. *Child Psychiatry Hum. Dev.* 44 591–601. 10.1007/s10578-012-0353-7 23247760

[B33] Heleniak C. Jenness J. L. Vander Stoep A. McCauley E. McLaughlin K. A. (2016). Childhood maltreatment exposure and disruptions in emotion regulation: a transdiagnostic pathway to adolescent internalizing and externalizing psychopathology. *Cogn. Ther. Res.* 40 394–415. 10.1007/s10608-015-9735-z 27695145 PMC5042349

[B34] Huang G. Su L. Su Q. Ren Y. (2006). An analysis of self-concept and family environment factors among children with oppositional defiant disorder. *Chin. J. Nervous Men. Dis.* 5 403–406.

[B35] Kim J. Cicchetti D. (2010). Longitudinal pathways linking child maltreatment, emotion regulation, peer relations, and psychopathology. *J. Child Psychol. Psychiatry* 51 706–716. 10.1111/j.1469-7610.2009.02202.x 20050965 PMC3397665

[B36] Ladd G. W. Profilet S. M. (1996). The child behavior scale: a teacher-report measure of young children’s aggressive, withdrawn, and prosocial behaviors. *Dev. Psychol.* 32 1008–1024. 10.1037/0012-1649.32.6.1008

[B37] Lansford J. E. Dodge K. A. Pettit G. S. Bates J. E. Crozier J. Kaplow J. (2002). A 12-year prospective study of the long-term effects of early child physical maltreatment on psychological, behavioral, and academic problems in adolescence. *Arch. Pediatr. Adoles. Med.* 156 824–830. 10.1001/archpedi.156.8.824 12144375 PMC2756659

[B38] Lavigne J. V. Dahl K. P. Gouze K. R. LeBailly S. A. Hopkins J. (2015). Multi-domain predictors of oppositional defiant disorder symptoms in preschool children: cross-informant differences. *Child Psychiatry Hum. Dev.* 46 308–319. 10.1007/s10578-014-0472-4 24997089 PMC4284149

[B39] Lavigne J. V. Gouze K. R. Hopkins J. Bryant F. B. LeBailly S. A. (2012). A multi-domain model of risk factors for ODD symptoms in a community sample of 4-year-olds. *J. Abnorm. Child Psychol.* 40 741–757. 10.1007/s10802-011-9603-6 22200893

[B40] Li L. Lin X. Chi P. Heath M. A. Fang X. Du H. (2016). Maltreatment and emotional and behavioral problems in Chinese children with and without oppositional defiant disorder: the mediating role of the parent–child relationship. *J. Interpers. Violence* 31 2915–2939. 10.1177/0886260515624234 26811315

[B41] Li Y. Hou X. Lin X. Wang Z. Fang X. Li J. (2016). The association of parents emotion dysregulation with oppositional defiant symptoms of migrant children: the role of parents conflict tactics and children emotion regulation. *Psychol. Dev. Educ.* 32 214–225. 10.16187/j.cnki.issn1001-4918.2016.02.11

[B42] Liao M. Lee A. S. Roberts-Lewis A. C. Hong J. S. Jiao K. (2011). Child maltreatment in China: an ecological review of the literature. *Child. Youth Serv. Rev.* 33 1709–1719. 10.1016/j.childyouth.2011.04.031 22194661

[B43] Lin X. Li L. Heath M. A. Chi P. Xu S. Fang X. (2016). Multiple levels of family factors and oppositional defiant disorder symptoms among Chinese children. *Fam. Process* 57 195–210. 10.1111/famp.12269 27900762

[B44] Lin X. Li L. Li Y. Wang Z. Chen Q. Fang X. (2014). How paternal and maternal psychological control affect on internalizing and externalizing problems of children with ODD symptoms. *Psychol. Dev. Educ.* 6 635–645. 10.16187/j.cnki.issn1001-4918.2014.06.010

[B45] Lindhiem O. Bennett C. B. Hipwell A. E. Pardini D. A. (2015). Beyond symptom counts for diagnosing oppositional defiant disorder and conduct disorder? *J. Abnorm. Child Psychol.* 43 1379–1387. 10.1007/s10802-015-0007-x 25788042 PMC4561600

[B46] Liu J. Cheng H. Leung P. W. (2011). The application of the preschool child behavior checklist and the caregiver–teacher report form to mainland chinese children: syndrome structure, gender differences, country effects, and inter-informant agreement. *J. Abnorm. Child Psychol.* 39 251–264. 10.1007/s10802-010-9452-8 20821258 PMC3042499

[B47] Liu X. Lin X. Zhou Q. Zhou N. Li Y. Lin D. (2017). Family and individual risk and protective factors of depression among Chinese migrant children with oppositional defiant disorder symptoms. *Front. Psychol.* 8:508. 10.3389/fpsyg.2017.00508 28421024 PMC5378708

[B48] Lovejoy M. C. Graczyk P. A. O’Hare E. Neuman G. (2000). Maternal depression and parenting behavior: a meta-analytic review. *Clin. Psychol. Rev.* 20 561–592. 10.1016/s0272-7358(98)00100-7 10860167

[B49] Morris A. S. Silk J. S. Steinberg L. Myers S. S. Robinson L. R. (2007). The role of the family context in the development of emotion regulation. *Soc. Dev.* 16 361–388. 10.1111/j.1467-9507.2007.00389.x 19756175 PMC2743505

[B50] Morris S. Z. Gibson C. L. (2012). Corporal punishment’s influence on children’s aggressive and delinquent behavior. *Crim. Justice Behav.* 38 818–839. 10.1177/0093854811406070

[B51] Muthén L. K. Muthén B. O. (1998-2017). *Mplus User’s Guide*, 5th Edn Los Angeles, CA: Muthén & Muthén.

[B52] Olson S. L. Tardif T. Z. Miller A. Felt B. Grabell A. S. Kessler D. (2011). Inhibitory control and harsh discipline as predictors of externalizing problems in young children: a comparative study of US, Chinese, and Japanese preschoolers. *J. Abnorm. Child Psychol.* 39 1163–1175. 10.1007/s10802-011-9531-5 21695446

[B53] Pardini D. A. Fite P. J. Burke J. D. (2008). Bidirectional associations between parenting practices and conduct problems in boys from childhood to adolescence: the moderating effect of age and African-American ethnicity. *J. Abnorm. Child Psychol.* 36 647–662. 10.1007/s10802-007-9162-z 17899362 PMC2981140

[B54] Preacher K. J. Hayes A. F. (2008). Asymptotic and resampling strategies for assessing and comparing indirect effects in multiple mediator models. *Behav. Res. Methods* 40 879–891. 10.3758/brm.40.3.879 18697684

[B55] Shields A. Cicchetti D. (1997). Reactive aggression among maltreated children: the contribution of attention and emotion dysregulation. *J. Clin. Child Psychol.* 27 381–395. 10.1207/s15374424jccp2704_2 9866075

[B56] Shields A. Cicchetti D. (2001). Parental maltreatment and emotion dysregulation as risk factors for bullying and victimization in middle childhood. *J. Clin. Child Psychol.* 30 349–363. 10.1207/S15374424JCCP3003_7 11501252

[B57] Shields A. Dickstein S. Seifer R. Giusti L. Magee K. D. Spritz B. (2001). Emotional competence and early school adjustment: a study of preschoolers at risk. *Early Educ. Dev.* 12 73–96. 10.1207/s15566935eed1201_5

[B58] Smith C. A. Farrington D. P. (2004). Continuities in antisocial behavior and parenting across three generations. *J. Child Psychol. Psychiatry* 45 230–247. 10.1111/j.1469-7610.2004.00216.x 14982238

[B59] Spinazzola J. Hodgdon H. Liang L. J. Ford J. D. Layne C. M. Pynoos R. (2014). Unseen wounds: the contribution of psychological maltreatment to child and adolescent mental health and risk outcomes. *Psychol. Trauma Theory Res. Pract. Policy* 6 S18–S28. 10.1037/a0037766

[B60] Steiner H. Remsing L. (2007). Practice parameter for the assessment and treatment of children and adolescents with oppositional defiant disorder. *J. Am. Acad. Child Adolesc. Psychiatry* 46 126–141. 10.1097/01.chi.0000246060.62706.af 17195736

[B61] Straus M. A. (1979). Measuring intrafamily conflict and violence: the conflict tactics (CT) scales. *J. Marriage Fam.* 41 75–88. 10.2307/351733

[B62] Straus M. A. Mickey E. L. (2012). Reliability, validity, and prevalence of partner violence measured by the conflict tactics scales in male-dominant nations. *Aggress. Violent Behav.* 17 463–474. 10.1016/j.avb.2012.06.004

[B63] Stringaris A. Goodman R. (2009). Longitudinal outcome of youth oppositionality: irritable, headstrong, and hurtful behaviors have distinctive predictions. *J. Am. Acad. Child Adolesc. Psychiatry* 48 404–412. 10.1097/CHI.0b013e3181984f30 19318881

[B64] Suveg C. Shaffer A. Morelen D. Thomassin K. (2011). Links between maternal and child psychopathology symptoms: mediation through child emotion regulation and moderation through maternal behavior. *Child Psychiatry Hum. Dev.* 42 507–520. 10.1007/s10578-011-0223-8 21484417

[B65] Tang A. M. Deng X. L. Du X. X. Wang M. Z. (2018). Harsh parenting and adolescent depression: mediation by negative self-cognition and moderation by peer acceptance. *Sch. Psychol. Int.* 39 22–37. 10.1177/0143034317709066

[B66] Tang C. Davis C. (1996). Child abuse in Hong Kong revisited after 15 years: characteristics of victims and abusers. *Child Abuse Neglect* 20 1213–1218. 10.1016/s0145-2134(96)00116-0 8985611

[B67] Thompson R. A. (1994). Emotion regulation: a theme in search of definition. *Monogr. Soc. Res. Child Dev.* 59 25–52. 10.2307/1166137 7984164

[B68] Trizano-Hermosilla I. Alvarado J. M. (2016). Best alternatives to Cronbach’s alpha reliability in realistic conditions: congeneric and asymmetrical measurements. *Front. Psychol.* 7:769. 10.3389/fpsyg.2016.00769 27303333 PMC4880791

[B69] Tung I. Lee S. S. (2014). Negative parenting behavior and childhood oppositional defiant disorder: differential moderation by positive and negative peer regard. *Aggress. Behav.* 40 79–90. 10.1002/ab.21497 23918473

[B70] Van der Oord S. Prins P. J. Oosterlaan J. Emmelkamp P. M. (2006). The association between parenting stress, depressed mood and informant agreement in ADHD and ODD. *Behav. Res. Ther.* 44 1585–1595. 10.1016/j.brat.2005.11.011 16405913

[B71] Wang M. Xing X. Zhao J. (2014). Intergenerational transmission of corporal punishment in china: the moderating role of marital satisfaction and gender. *J. Abnorm. Child Psychol.* 42 1–12. 10.1007/s10802-014-9890-9 24915779

[B72] Xu Y. Farver J. A. M. Zhang Z. (2009). Temperament, harsh and indulgent parenting, and Chinese children’s proactive and reactive aggression. *Child Dev.* 80 244–258. 10.1080/01650250500147121 19236404

[B73] Zhao X. Zhang Y. Li L. Zhou Y. (2005). Evaluation on reliability and validity of Chinese version of childhood trauma questionnaire. *Chin. J. Clin. Rehabil.* 9 209–211. 10.1371/journal.pone.0208779 30543649 PMC6292582

[B74] Zhao Y. (2013). *Family Conflict Characteristics of Chinese Oppositional Defiant Disorder Children: A Qualitative Study.* Bachelor’s dissertation, Beijing Normal University: Beijing.

