# Corrigendum: Family Risk Factors Associated With Oppositional Defiant Disorder Symptoms, Depressive Symptoms, and Aggressive Behaviors Among Chinese Children With Oppositional Defiant Disorder

**DOI:** 10.3389/fpsyg.2020.00131

**Published:** 2020-02-06

**Authors:** Xiuyun Lin, Yanbin Li, Shousen Xu, Wan Ding, Qing Zhou, Hongfei Du, Peilian Chi

**Affiliations:** ^1^Institute of Developmental Psychology, Beijing Normal University, Beijing, China; ^2^Beijing Key Laboratory of Applied Experimental Psychology, School of Psychology, Beijing Normal University, Beijing, China; ^3^Department of Psychology, College and Graduate School of Arts and Sciences, University of Virginia, Charlottesville, VA, United States; ^4^School of Kinesiology and Health, Capital University of Physical Education and Sports, Beijing, China; ^5^Department of Psychology, University of California, Berkeley, Berkeley, CA, United States; ^6^Department of Psychology, Guangzhou University, Guangzhou, China; ^7^Department of Psychology, University of Macau, Macau, China

**Keywords:** parent emotion dysregulation, harsh parenting practices, oppositional defiant disorder, child depressive symptoms, child aggressive behaviors, descriptive survey study

There is an error in the Funding statement. The correct number for **National Key R&D Program of China is 2018YFF0300603**. The correct number for **Training Program for Young Top Talents in Beijing-affiliated Universities is CIT&TCD201504085**. The correct number for **National Natural Science Foundation of China is 31800935**.

In addition, there is an error in the Funding statement. The correct Names for the Funder are **National Key R&D Program of China, Training Program for Young Top Talents in Beijing-affiliated Universities, and National Natural Science Foundation of China**. The corrected funding statement appears below.

## Funding

The study described in this report was funded by National Key R&D Program of China (2018YFF0300603), Training Program for Young Top Talents in Beijing-affiliated Universities (CIT&TCD201504085), and National Natural Science Foundation of China (31800935).

The authors apologize for this error and state that this does not change the scientific conclusions of the article in any way. The original article has been updated.

